# Discomfort associated with fixed orthodontic appliances: determinant factors and influence on quality of life

**DOI:** 10.1590/2176-9451.19.3.102-107.oar

**Published:** 2014

**Authors:** Leandro Silva Marques, Saul Martins Paiva, Raquel Gonçalves Vieira-Andrade, Luciano José Pereira, Maria Letícia Ramos-Jorge

**Affiliations:** 1 Adjunct professor, Federal University of the Jequitinhonha and Mucuri Valleys, UFVJM.; 2 Full professor, Federal University of Minas Gerais, UFMG.; 3 PhD Resident, Department of Paediatric Dentistry, School of Dentistry, Federal University of Minas Gerais, UFMG.; 4 Associate professor, Department of Physiology and Pharmacology, School of Dentistry, Federal University of Lavras, UFLA.; 5 Professor, Federal University of the Jequitinhonha and Mucuri Valleys, UFVJM.

**Keywords:** Adolescent, Orthodontics, Quality of life

## Abstract

**Objective:**

To investigate the determinant factors of discomfort attributed to the use of fixed orthodontic appliance and the effect on the quality of life of adolescents.

**Material and Methods:**

Two hundred and seventy-two individuals aged between 9 and 18 years old, enrolled in public and private schools and undergoing orthodontic treatment with fixed appliance participated in this cross-sectional study. The participants were randomly selected from a sample comprising 62,496 individuals of the same age group. Data was collected by means of questionnaires and an interview. Discomfort intensity and bio-psychosocial variables were assessed using the Oral Impact on Daily Performance questionnaire. Self-esteem was determined using the Global Negative Self-Evaluation questionnaire. Statistical analysis involved the chi-square test and both simple and multiple Poisson regression analyses.

**Results:**

Although most individuals did not present discomfort, there was a prevalence of 15.9% of impact on individuals' daily life exclusively due to the use of fixed orthodontic appliance . Age [PR: 3.2 (95% CI: 1.2-8.5)], speech impairment [PR: 2.2 (95% CI: 1.1-4.6)], poor oral hygiene [PR: 2.4 (95% CI: 1.2-4.8)] and tooth mobility [PR: 3.9 (95% CI: 1.8-8.1)] remained independently associated with a greater prevalence of discomfort (P ≤ 0.05).

**Conclusions:**

Discomfort associated with the use of fixed orthodontic appliances exerted a negative influence on the quality of life of the adolescents comprising the present study. The determinants of this association were age, poor oral hygiene, speech impairment and tooth mobility.

## Abstract (pt)

**Objetivo:**

investigar os fatores determinantes do desconforto atribuído ao uso do aparelho ortodôntico fixo e sua influência na qualidade de vida de adolescentes.

**Métodos:**

participaram desse estudo transversal 272 indivíduos, com idades entre 9 e 18 anos, estudantes de escolas públicas e privadas, que usavam aparelhos ortodônticos fixos (braquetes). Os participantes foram selecionados aleatoriamente, entre 62.496 indivíduos da mesma faixa etária. A coleta de dados foi feita em forma de entrevista e questionário. A intensidade do desconforto e variáveis biopsicossociais foram avaliadas por meio do *Oral Impact on Daily Performance* (OIDP). A autoestima foi determinada pela *Global Negative Self-Evaluation* (GSE). A análise estatística envolveu o teste qui-quadrado e a análise de regressão de Poisson, simples e múltipla.

**Resultados:**

embora a maioria dos indivíduos não apresentasse desconforto, observou-se uma prevalência de impacto na vida diária devido, exclusivamente, ao uso do aparelho ortodôntico fixo, de 15,9%. Variáveis idade de 15 a 18 anos [RP = 3,2 (IC 95% = 1,2-8,5)], dificuldade de falar [RP = 2,2 (IC 95% = 1,1-4,6)], dificuldade de limpar a boca [RP = 2,4 (IC95% = 1,2-4,8)] e mobilidade dos dentes [RP = 3,9 (IC 95% = 1,8-8,1)] permaneceram associadas, de forma independente, à maior prevalência de desconforto (p ≤ 05).

**Conclusões:**

desconforto associado ao uso de aparelhos ortodônticos fixos influenciou negativamente a qualidade de vida de adolescentes. Os fatores determinantes foram idade, dificuldade de limpar a boca, de falar e mobilidade dentária.

## INTRODUCTION

Orthodontic treatment can be an uncomfortable experience. Orthodontic appliances are foreign objects inserted into a sensitive area of the body, causing both physical and psychological discomfort.^1^ Such discomfort can exert a negative influence on patient's desire to undergo treatment, cooperation and quality of treatment itself.^2^ The main factors associated with the discomfort experienced by orthodontic patients are: The type of appliance, amount of force applied in the early stages of treatment, previous experiences with pain and emotional, cognitive and environmental aspects such as culture, sex and age.^2-10^

Thus, depending on the stage, orthodontic treatment may negatively influence patients' quality of life.^11,12^ Feu et al^13^ have recently conducted a longitudinal study in which they found that patients who had already removed the braces had better indicators of quality of life than those who were still in treatment.^13^

While most studies focus on the operator's point of view, addressing variables such as patient cooperation and adaptability to orthodontic treatment,^2-9^ little attention has been given to patients' perception with regard to discomfort.^10^ Moreover, a critical analysis of the literature reveals that most studies on patient discomfort during orthodontic treatment address aspects related to pain. Thus, a large number of other variables related to patient's physical and psychological well-being have not been considered. A possible explanation is the fact that most studies do not use adequate tools for assessing the specific impact of the use of an orthodontic appliance on patient's quality of life.

The aim of the present study was to investigate the determinant factors of discomfort attributed to the use of a fixed orthodontic appliance and the effect on the quality of life of adolescents.

## MATERIAL AND METHODS

This cross-sectional study was carried out in the Brazilian state of Minas Gerais which has 853 municipalities and is the second most populous state in Brazil, with approximately 20 million inhabitants. Participants were selected from a population of 62,496 individuals aged between 9 and 18 years old. The following inclusion criterion was applied: patient undergoing orthodontic treatment with fixed appliances (braces) for at least six months.

Data were collected from public and private schools of ten different cities (one public school and one private school per city, totaling 20 schools) between October and December, 2010. Both schools and individuals were randomly selected by lots. The number of participants from each city and school was proportional to the actual distribution considering the total number of individuals in this age group residing in the ten cities surveyed. Calculation of sample size was performed using a standard error of 5% or less, 95% confidence interval and 20% prevalence of discomfort associated with the use of orthodontic fixed appliance. The minimal sample size was determined in 246 individuals, to which 20% was added to compensate for potential losses, thus totaling 295 participants.

Discomfort attributed specifically to the use of orthodontic fixed appliances was the dependent variable considered for this study. It was recorded through the Oral Impact on Daily Performances (OIDP) questionnaire answered in the form of an interview.^10^ Therefore, any factor associated with another type of discomfort was not included and cannot be considered as a confounding factor. Possible confounding factors such as caries, trauma, gingivitis etc., were controlled, since individuals who reported discomfort associated with some of these conditions did not participate in the statistical analysis of the study. The OIDP is an instrument used to record the impact of oral conditions on an individual's capacity to perform daily activities in the previous six months. This instrument is objective and addresses the main bio-psychosocial consequences of dental problems. For quantification of this variable, the participants were classified as either not experiencing discomfort or experiencing discomfort. Moreover, the total impact per participant was determined considering the frequency and severity of discomfort.

Global Negative Self-Evaluation (GSE) questionnaire was used to assess patient's self-esteem^11^. GSE is a six-item scale. Each item has six response options which are quantified in increasing order from 1 to 6. For classification of self-esteem, the responses of each item are summed up and the result is divided by six, thereby obtaining a self-esteem value which is dichotomized as low self-esteem (scores between 1 and 2.69) and high self-esteem (scores between 2.7 and 6).^10^

Bivariate analysis (chi-square test) was first performed to determine associations between patient-reported discomfort due to the use of a fixed orthodontic appliance and demographic/ bio-psychosocial variables as well as the specific causes of discomfort (P ≤ 0.05). The variables that demonstrated a significant association with discomfort were then incorporated into Poison model with robust variance to determine independent associations between predictor variables and discomfort due to the use of a fixed orthodontic appliance. The magnitude of association between each factor and discomfort was determined based on unadjusted and adjusted prevalence ratios (PR), respective confidence intervals (CI = 95%) and p-values (Wald test).

A letter was sent to the adolescents' parents/guardians requesting their participation and explaining the characteristics and importance of the study. An informed consent was required, with no negative consequences for those who refused to participate. This study was approved by the Institutional Review Board of the University Green River Valley (UNINCOR), Brazil.

## RESULTS

Two hundred seventy-two individuals participated in the present study (92% response rate). Males accounted for 36% of the sample. The prevalence of impact on daily activities exclusively due to the use of a fixed orthodontic appliance was 15.9%. Only 17.6% of the interviewees reported no impact associated with oral health. The OIDP scores ranged from 0 to 78.5 (mean: 12.8, standard deviation: 9.6; median: 11.6). Among the impacts, 38% were categorized as low-intensity, 20.2% were categorized as moderate-intensity and 13.6% were categorized as severe and very severe-intensity.

Discomfort attributed to the use of fixed orthodontic appliance was significantly associated with difficulties in eating, oral hygiene and speech, as well as tooth mobility, halitosis, impaired taste, and bleeding gingiva (Tables 1 and 2).

**Table 1:** Association between discomfort reported by patients,due to the use of fixed orthodontic appliance, and demographic/bio-psychosocial variables.

Discomfort – use of orthodontic appliance			
	Absent n (%)	Present n (%)	*p*
Age			
9 to 14 years	110 (95.7)	5 (4.3)	< 0.001^C^
15 to 18 years	119 (80.4)	29 (19.6)	
Sex			
Male	83 (84.7)	15 (15.3)	0.294^C^
Female	155 (89.1)	19 (10.9)	
Difficulty in eating			
No	156 (93.4)	11 (6.6)	< 0.001^C^
Yes	82 (78.1)	23 (21.9)	
Speech impairment			
No	148 (92.5)	12 (7.5)	0.003^C^
Yes	90 (80.4)	22 (19.6)	
Poor oral hygiene			
No	159 (93.0)	12 (7.0)	< 0.001^C^
Yes	79 (78.2)	22 (21.8)	
Difficulty in showing the teeth			
No	185 (87.3)	27 (12.7)	0.825^C^
Yes	53 (88.3)	7 (11.7)	
Difficulty in sleeping			
No	182 (88.3)	24 (11.7)	0.454^C^
Yes	56 (84.8)	10 (15.2)	
Difficulty in maintaining a stable emotional state			
No	201 (87.0)	30 (13.0)	0.564^C^
Yes	37 (90.2)	4 (9.8)	
Difficulty in performing school tasks			
No	198 (85.7)	33 (14.3)	0.035^C^
Yes	40 (97.6)	1 (2.4)	
Difficulty to get along with friends			
No	217 (86.8)	33 (13.2)	0.330^F^
Yes	21 (95.5)	1 (4.5)	

C Pearson's chi-square test

F Fisher's exact test

**Table 2:** Bivariate analysis of association between discomfort reported by patients due to the use of fixed orthodontic appliance and specific causes of discomfort

Discomfort – use of orthodontic appliance			
	Absent n (%)	Present %)	*p*
Pain			
No	171 (85.5)	29 (14.5)	0.096^C^
Yes	67 (93.1)	5 (6.9)	
Missing tooth			
No	230 (87.5)	33 (12.5)	1.000^F^
Yes	8 (88.9)	1 (11.1)	
Tooth mobility			
No	228 (90.8)	23 (9.2)	< 0.001^F^
Yes	10 (47.6)	11 (52.4)	
Position of teeth			
No	224 (88.2)	30 (11.8)	0.257^F^
Yes	14 (77.8)	4 (22.2)	
Shape/size of teeth			
No	228 (87.4)	33 (12.6)	1.000^F^
Yes	10 (90.9)	1 (9.1)	
Deformity of mouth/face			
No	236 (88.1)	32 (11.9)	0.078^F^
Yes	2 (50.0)	2 (50.0)	
Blisters			
No	182 (87.1)	27 (12.9)	0.704^C^
Yes	56 (88.9)	7 (11.1)	
Sensation of dry mouth			
No	220 (86.6)	34 (13.4)	0.141^F^
Yes	18 (100)	0	
Halitosis			
No	226 (90.8)	23 (9.2)	< 0.001^C^
Yes	12 (52.2)	11 (47.8)	
Impaired taste			
No	235 (89.7)	27 (103)	< 0.001^F^
Yes	3 (30)	7 (70)	
Halitosis			
No	226 (89.7)	26 (10.3)	0.001^F^
Yes	12 (60.0)	8 (40.0)	
Gingival bleeding			
No	220 (92.1)	19 (7.9)	< 0.001^F^
Yes	18 (54.5)	15 (45.5)	
Gingival recession			
No	235 (87.4)	34 (12.6)	1.000^F^
Yes	3 (100)	0	
Difficulty opening the mouth			
No	237 (87.5)	34 (12.6)	1.000^F^
Yes	1 (100.0)	0	
Self-esteem (n=271)			
High	215 (88.5)	28 (11.5)	0.137^F^
Low	22 (78.6)	6 (21.4)	

C Pearson's chi-square test

F Fisher's exact test.

## DISCUSSION

The results of the present study demonstrate that approximately 16% of participants experienced discomfort due to the use of a fixed orthodontic appliance, which exerted a negative influence on their quality of life. Thus, identifying determinants of this discomfort and understanding how such factors affect the physical and psychological well-being of individuals who wear braces can enhance the odds of achieving successful orthodontic treatment.^4^

Perception and intensity of discomfort are directly associated with the individual characteristics of each patient, namely: self-esteem, self-confidence, treatment compliance, expectations, perception of dental esthetics and severity of the malocclusion.^4,16^ Self-esteem did not affect the occurrence of discomfort in the present study, but the literature offers evidence that self-confidence may be affected not only by limitations to speech, but also to visibility of braces, especially during social interactions in which attention is focused on the face and mouth.^4^ It is noteworthy that adolescent patients whose reasons for seeking orthodontic treatment are mainly guided by the perception of their appearance feel more as the center of attention among their friends and acquaintances during treatment.^8,15,16,17^

An interesting, unexpected finding of the present study was the lack of association between pain and discomfort. Tooth movement produced by orthodontic appliances causes discomfort and it has been reported that the fear of pain is a key factor dissuading patients from seeking orthodontic treatment.^18^ On the other hand, pain during treatment gradually increases within 4 to 24 hours after adjusting the appliance, but returns to normal by the seventh day.^2,19,20,21^ Thus, patients can adapt to pain and discomfort with the progression of treatment, as the sensations either stop completely or at least cease to be the focus of attention. This is a possible explanation for the fact that patients of the present study did not cite pain as exerting a negative influence on their quality of life. Moreover, all participants had been undergoing orthodontic treatment for at least six months. This finding could favor the clinical conduct of orthodontists, as future patients should be informed about how and to what extent orthodontic treatment can affect their physical and psychological well-being.

Another aspect concerning pain is the magnitude of force associated with the orthodontic arch, especially during the early phases of treatment. Classical histological studies suggest that light forces are more biologically efficient and less traumatic during orthodontic tooth movement.^12,16^ Moreover, the greater the degree of initial crowding, the more teeth will be actively incorporated by the orthodontic archwire and the greater the potential for high degrees of force.^2^

Younger patients demonstrated greater tolerance and adaptation to discomfort caused by fixed orthodontic appliances. This result corroborates findings described in previous studies,^3,20,21^ but differs from the findings reported in another study.^2^ Such differences may be related to cultural aspects and study design.

Poor oral hygiene, speech impairment and tooth mobility also exerted a negative effect on the daily activities of participants.^17^ These findings demonstrate that orthodontists should not overlook the discomfort experienced by wearers of fixed orthodontic appliances and highlight the need for effective orthodontist-patient communication.

Confounding factors, such as blisters, caries and halitosis, were controlled. The dependent variable was discomfort exclusively associated with the use of fixed appliances. Individuals who reported more than one type of discomfort besides the use of the appliance were not included in the statistical model. Further studies, especially those of prospective nature, are necessary to investigate the critical stages of the negative interference of the use of the appliance on the quality of life of individuals.

## CONCLUSIONS

1. Discomfort associated with the use of fixed orthodontic appliances exerted a negative influence on the quality of life of the adolescents of the present study.
2. The determinants of this association were: Age, poor oral hygiene, speech impairment and tooth mobility.

## Figures and Tables

**Table 3:** Simple and multiple Poisson regression analysis of association between discomfort due to the use of orthodontic appliance, and bio-psychosocial variables.

	Unadjusted Rate ratio			Adjusted Rate ratio		
	PR	(CI 95%)	p*	PR	(CI 95%)	p**
Age (n = 263)						
9 to 14 years	1.0	(1.8; 11.7)	0.002	1.0	(1.2; 8.5)	0.020
15 to 28 years	4.5			3.2		
Difficulty in eating						
No	1.0	(1.6; 6.7)	0.001			
Yes	3.3					
Speech impairment						
No	1.0	(1.2-4.9)	0.012	1.0	(1.1; 4.6)	0.032
Yes	2.4			2.2		
Poor oral hygiene						
No	1.0	(1.6; 6.4)	0.001	1.0	(1.2; 4.8)	0.018
Yes	3.2			2.4		
Difficulty in performing school tasks						
No	1.0	(0.0; 1.2)	0.074			
Yes	0.2					
Pain						
No	1.0	(0.2; 1.3)	0.143			
Yes	0.5					
Tooth mobility						
No	1.0	(2.7; 11.3)	< 0.001	1.0	(1.8; 8.1)	< 0.001
Yes	5.5			3.9		
Halitosis						
No	1.0	(2.4; 10.2)	< 0.001			
Yes	4.9					
Impaired taste						
No	1.0	(1.8; 8.7)	0.001			
Yes	3.9					
Gingival bleeding						
No	1.0	(2.8; 10.8)	< 0.001			
Yes	5.5					
Deformity of mouth/face						
No	1.0	(0.9; 16.8)	0.056			
Yes	4.0					
Self-esteem (n=271)						
High	1.0	(0.7; 4.3)	0.195			
Low	1.8					

* Simple Poisson regression

** Multiple Poisson regression.
